# Green synthesis of copper & copper oxide nanoparticles using the extract of seedless dates

**DOI:** 10.1016/j.heliyon.2019.e03123

**Published:** 2020-01-30

**Authors:** Elwy A. Mohamed

**Affiliations:** Agricultural Research Center, P.O. Box 12619, Giza, Egypt

**Keywords:** Materials science, Chemistry, Copper nanoparticles, Green synthesis, Date palm, Seedless dates, Chemical reduction

## Abstract

In the last few years, copper and copper oxide nanoparticles were involved in many applications; this encouraged many researchers worldwide to develop more facile synthesis methods. Unprecedentedly, the current study reports a green method for synthesizing ***copper/copper oxide nanoparticles*** (Cu/Cu_2_O NPs) using the extract of seedless dates. Cu/Cu_2_O NPs were synthesized according to the chemical reduction method using seedless dates' extract as a reducing agent due to its high content of phenolics and flavonoids. Transmission Electron Microscopy (TEM) revealed that roughly spherical particles were synthesized. Dynamic Light Scattering (DLS) showed that the synthesized Cu/Cu_2_O NPs have an average particle size of 78 nm and zeta potential of +41 mV, indicating a good stability of the particles. Successful synthesis of Cu/Cu_2_O NPs was affirmed through both X-Ray Diffraction (XRD), which revealed the presence of the characteristic peaks of copper at 2θ = 43.2745, 50.4083 and 74.1706°, and UV-Vis. Spectroscopy, which revealed the surface plasmonic resonance peak characterizes Cu/Cu_2_O NPs at 576 nm. In addition, Fourier Transform Infrared Spectroscopy (FTIR) revealed the presence of phenolic compounds, which were responsible for reducing copper ions into copper nanoparticles through their carbonyl and hydroxyl linkages, adsorbed from the extract on Cu/Cu_2_O NPs. Conclusively, the current work provides, for the first time, a simple, cost-effective and environmentally friendly method for synthesizing Cu/Cu_2_O NPs using useless seedless dates.

## 1 Introduction

Since Richard Feynman's inspiring talk on December 29, 1959 titled "*There's Plenty of Room at the Bottom*" at the annual American Physical Society meeting at California Institute of Technology (Caltech) [1], thousands of researchers all over the world began to explore and exploit the versatile chances offered by nanotechnology generally and unique properties of nanoparticles specifically in different fields. Among various types of nanoparticles, copper and copper oxide nanoparticles attract much attention because of their distinguished catalytic, mechanical, magnetic, electric and thermal properties; in addition to their versatile applicability in many fields including agricultural, industrial, environmental and medical applications [2]. Furthermore, copper and copper oxide nanoparticles can be used in catalysis [3], sensors [4], degradation of dyes [5], fungicidal [6, 7] and nematicidal [8] applications.

Synthesis methodologies are considered of utmost importance in the field of nanotechnology. In this regard, the methodologies which have adopted to synthesize Cu/Cu_2_O NPs vary among three main categories; physical, biological and chemical methods [9]. The core idea beyond synthesis of metallic nanoparticles generally, and copper nanoparticles particularly, depends in its simplest forms on providing three main components; a precursor to provide copper ions, a reducing agent to provide electrons required to reduce the copper ions into copper atoms which then aggregated into copper nanoparticles with a limited size under the control of the third component, the surfactant; under the optimal pH and temperature conditions.

When the source of electrons, i.e. the reducing agent, is a chemical compound, the method is chemical [10, 11]; while when the source of electrons is a physical source such as the electric current, the method is physical [12], and when the source of electrons is an organism then the method is biological [13].

Chemical methods can be categorized into two fundamental classes, traditional and green chemical methods. Traditional chemical methods usually use toxic synthetic chemicals as a reducing agent, such as sodium borohydride [10], hypophosphite [14] and Hydrazine [11] … etc. while green chemical methods usually use natural chemicals as a reducing agent, such as citric acid [15] and ascorbic acid [16]. Green chemical methods surplus over traditional methods; because they are usually nontoxic, ecofriendly and more cost-effective.

Currently, many methodologies usually use toxic reducing agents or complex procedures, which consume high amounts of energy [17]. Thus many researchers were encouraged to develop simpler procedures using less toxic or even non-toxic chemicals [18].

In this regard, many papers have reported the usage of different plant extracts to prepare copper and copper oxide nanoparticles such as *Nerium oleander* Leaf aqueous extract [19], peel extract of *Punica granatum* [20], fruit extract of *Ziziphus spina-christi* L. [21], *Rosa canina* fruit extract [22], fruit extract of *Syzygium alternifolium* (Wt.) Walp [23] and *Asparagus adscendens* Roxb. root and leaf extract [24], some of which might be not cost effective or not easily available.

This paper reports, for the first time, the synthesis of copper and copper oxide nanoparticles using the extract of seedless dates, which are dates without seeds due to incomplete fertilization, as a reducing agent. Since the unfertilized dates are considered of relatively low or even no economic value and are easily available [25], the usage of seedless dates has the advantages of being cost effective and environment-friendly.

## 2 Materials and methods

### 2.1 Chemicals

All the utilized chemicals were of analytical purity standard and were used as provided.

For synthesizing Cu/Cu_2_O NPs, Cetyl trimethylammonium bromide (CTAB) (Sigma-Aldrich, Egypt), Copper sulfate pentahydrate (Elnasr Pharmacuticals Co., Egypt) and Ethanol (Sigma-Aldrich, Egypt) were used.

### 2.2 Instrumentation

The used instruments include Electric Blender (Monolex), Centrifugation Machine (Hettich centrifuge), pH Meter (Jenway 3510), Hotplate Stirrer (Stuart), Vortex (IKA), X-Ray Diffractometer, (Philips PW1840 X-Ray Diffractometer, USA), FTIR Spectrophotometer (Jasco 4100, Japan; 400–4000 cm^−1^), UV-Vis. Spectrophotometer (Helios Gamma Spectrophotometer), Transmission Electron Microscope (Tecnai G20, Super twin, double tilt, FEI, Netherland), and Dynamic Light Scattering Machine (Zetasizer nano series (Nano ZS), Malvern, UK).

### 2.3 Preparation of the extract from seedless dates

1 kg of freshly excised seedless dates, Samany cultivar, was washed and homogenized well with 1 L of deionized water in an electric blender. After that, the resulted mixture was filtered through centrifugation at 4000 rpm for 10 min, the pellet was discarded and the supernatant was used as it is without any further purification as a reducing agent. The supernatant was a pale yellow liquid.

### 2.4 Synthesis of Cu/Cu_2_O NPs

Synthesis of Cu/Cu_2_O NPs was done according to the chemical reduction method [16] with a modification in which the ascorbic acid was replaced with the seedless dates extract. To 100 mL of the seedless dates extract, 1 gm of Cetyl trimethylammonium bromide (CTAB) was added under stirring, then the pH of the mixture was adjusted at 6.8 and the mixture's temperature was elevated to 80 °C; after that, 0.1 g of copper sulfate pentahydrate was dissolved in 10 mL deionized water and was added drop by drop under stirring to the mixture of the extract and CTAB. The reaction was continued under stirring until a reddish brown color was developed indicating the successful preparation of Cu/Cu_2_O NPs.

The synthesized Cu/Cu_2_O NPs were collected centrifugally at 4000 rpm for 5 min, supernatant (reaction medium) was discarded and the pellet (Cu/Cu_2_O NPs) was resuspended in deionized water using vortex for washing. Then, Cu/Cu_2_O NPs were collected again centrifugally at 4000 rpm for 5 min. Washing with deionized water was repeated three times through consecutive precipitation and resuspension in deionized water, and then washing with absolute ethanol was repeated three times through consecutive precipitation and resuspension in absolute ethanol. Finally, Cu/Cu_2_O NPs were air-dried and collected for further characterization.

### 2.5 Characterization of Cu/Cu_2_O NPs

To confirm successful synthesis of Cu/Cu_2_O NPs, X-Ray Diffraction (XRD) was performed on powder Cu/Cu_2_O NPs using X-Ray diffractometer.

In addition, to investigate the interaction of the extract with copper sulfate pentahydrate, Fourier Transform Infrared Spectroscopy (FTIR) was performed also on powder Cu/Cu_2_O NPs using FTIR spectrophotometer (400–4000 cm^−1^).

Furthermore, the characteristic surface plasmon resonance of Cu/Cu_2_O NPs was detected using UV-Vis. Spectrophotometer; and Transmission Electron Microscopy was used to figure out the shape of the synthesized Cu/Cu_2_O NPs.

Also, Dynamic light scattering (DLS) was used to determine both the Particles Size Distribution (PSD) and Zeta Potential (ZP) of the synthesized Cu/Cu_2_O NPs, with the Standard Operating Procedure of the DLS instrument.

## 3 Results and discussion

### 3.1 X-ray diffraction (XRD)

**Figure 1:**
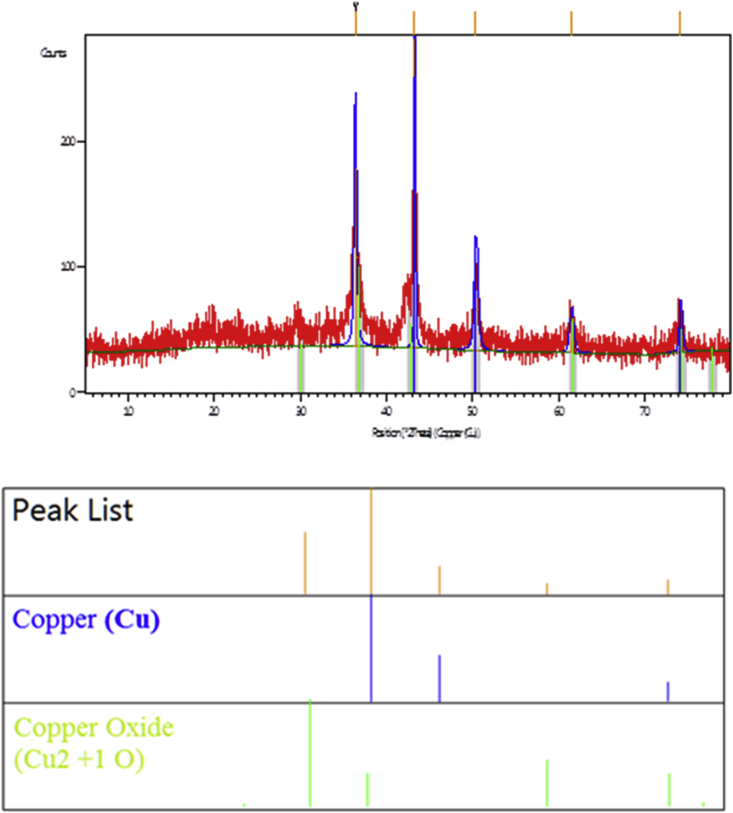
X-Ray Diffraction (XRD) pattern of the synthesized Cu/Cu_2_O NPs, as obtained from the X-Ray Diffractometer, showing the main diffraction peaks characterize the elemental copper at 2θ = 43.2745, 50.4083 and 74.1706°, in addition to those characterize Cu_2_O at 2 θ = 36.3967 and 61.4835°.

X-Ray Diffraction pattern, as shown in Figure 1, confirmed the successful synthesis of copper nanoparticles with a shell of copper oxide; wherein the main diffraction peaks characterize the elemental copper were detected at 2θ = 43.2745, 50.4083 and 74.1706° which correspond to the **(1 1 1)**, **(2 0 0)**, and **(2 2 0)** crystal faces of copper [26]. It is also noteworthy that there are other peaks at 2θ = 36.3967 and 61.4835°, that are characteristic for Cu_2_O; both peaks were attributed to the presence of a Cu_2_O shell covering the copper core [27].

Noteworthy, the XRD pattern of the prepared nanoparticles contains the characteristic peaks of both copper and copper oxide. This mixed phase structure is not attributed to the air-drying of the prepared nanoparticles, since Mustafa Biçer & İlkay Şişman reported that the XRD pattern of the freshly prepared copper nanoparticles contains the same peaks as those exhibited by the XRD pattern of the same copper nanoparticles sample after being exposed to the air for 24 h. At the same time, Mustafa Biçer & İlkay Şişman reported that the characteristic peak of copper oxide was emerged only through decreasing the reaction temperature from 85 °C to 60 °C; since at 60 °C the reducing agent (ascorbic acid in that case) could not completely reduce Cu^2+^ into Cu^0^ atoms, thus the characteristic peaks of copper oxide were emerged; Unlike at 85 °C, the reducing agent could completely reduce Cu^2+^ into Cu^0^ atoms, thus the characteristic peaks of copper oxide were not emerged [16]. It is reasonable that each reducing agent has its own temperature at which it can completely reduce Cu^2+^ into Cu^0^ atoms. In the current work, the reaction temperature of 80 °C was chosen so as not to enable the reducing agent (the seedless dates extract in this case) to completely reduce Cu^2+^ into Cu^0^ atoms, hence producing the mixture of both copper and copper oxide in the same particle (i.e. Cu/Cu_2_O NPs).

List of peaks and their respective positions [°2θ] for the synthesized Cu/Cu_2_O NPs as obtained from the X-Ray Diffractometer are shown in Table 1.

**Table 1:** List of peaks and their respective positions [°2θ] for the synthesized Cu/Cu_2_O NPs as obtained from the X-Ray Diffractometer.

No.	Pos. [°2θ]	Height [cts]	d-spacing [Å]	Rel. Int. [%]	Crystallite Size only [Å]	Micro Strain only [%]
1.	36.3967	142.23	2.46852	57.65	270.475500	0.456329
2.	43.2745	246.71	2.09080	100.00	968.194900	0.107974
3.	50.4083	64.06	1.81037	25.97	211.491000	0.428002
4.	61.4835	26.61	1.50818	10.79	222.752100	0.338534
4.	74.1706	35.04	1.27850	14.20	323.138300	0.197825

### 3.2 Fourier transform infrared spectroscopy (FTIR)

**Figure 2:**
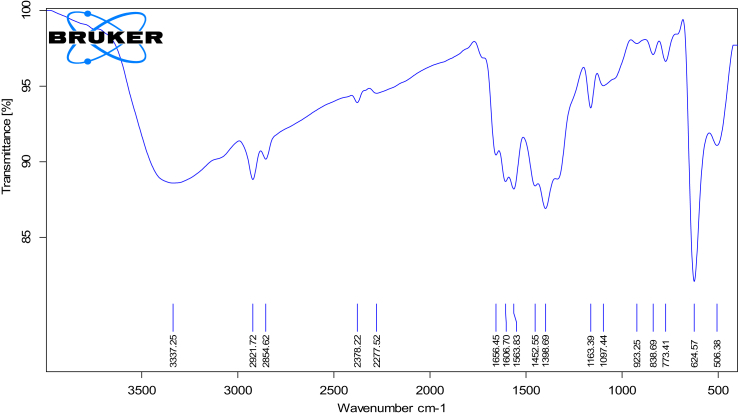
FTIR spectrum of the synthesized Cu/Cu_2_O NPs showing the absorption peaks of the functional groups adsorbed on the synthesized Cu/Cu_2_O NPs at 3337.25; 2921.72; 1656.45; 1606.70; 1452.55; 1398.69; 1163.39 and 1097.44 cm^−1^, which represent O–H stretching vibrations (alcoholic or phenolic), C–H asymmetric stretching, C=C stretching, C=C stretching, C=C aromatic ring stretching, C–OH stretching vibrations, C–OH bending and C–OH bending, respectively.

FTIR spectrum, as shown in Figure 2, of the synthesized Cu/Cu_2_O NPs revealed the presence of eight main peaks at 3337.25; 2921.72; 1656.45; 1606.70; 1452.55; 1398.69; 1163.39 and 1097.44 cm^−1^, which represent O–H stretching vibrations (alcoholic or phenolic), C–H asymmetric stretching, C=C stretching, C=C stretching, C=C aromatic ring stretching, C–OH stretching vibrations, C–OH bending and C–OH bending, respectively; as shown in Table 2. These peaks affirmed the adsorption of phenolic compounds from the seedless dates extract on the surface of the prepared nanoparticles via the interaction of π electrons [28]. Furthermore, the reduction of copper ions into copper nanoparticles was achieved under the effect of hydroxyl and carbonyl linkages in the extract's constituents [29]. In addition, since phenolic compounds were adsorbed on the surface of the nanoparticles, then the phenolic compounds may act as a capping agent, thus provide the nanoparticles with more stability. The prepared nanoparticles were stable over 24 h.

**Table 2:** The absorption peaks of the prepared Cu/Cu_2_O nanoparticles as obtained from FTIR-spectrophotometer and their corresponding functional groups.

No.	Absorption Peak Position (Wavenumber) (Cm^−1^)	Functional Group
1.	3337.25	O–H stretching vibrations (alcoholic or phenolic)
2.	2921.72	C–H asymmetric stretching
3.	1656.45	C=C stretching
4.	1606.70	C=C stretching
5.	1452.55	C=C aromatic ring stretching
6.	1398.69	C–OH stretching vibrations
7.	1163.39	C–OH bending
8.	1097.44	C–OH bending

In addition, the presence of the characteristic vibrational peak of Cu_2_O is observed at 624.57 cm^−1^ [30,31], which agree with the XRD results.

### 3.3 UV-vis. Spectroscopy

**Figure 3:**
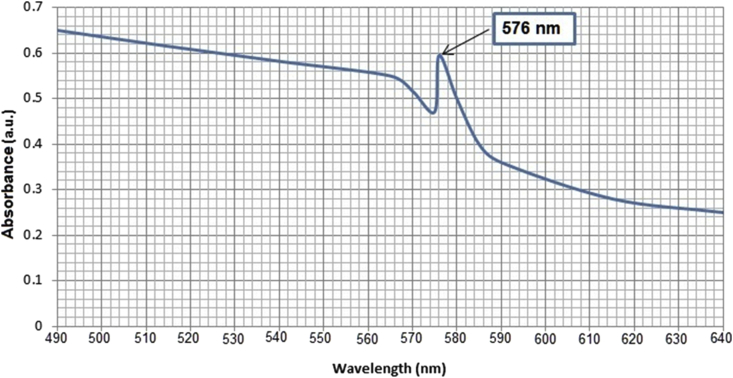
UV-Vis. Spectrogram of the synthesized Cu/Cu_2_O NPs showing their characteristic surface plasmonic resonance (SPR) at 576 nm.

UV-Vis. Spectrogram, as shown in Figure 3, depicted that Cu/Cu_2_O NPs were synthesized successfully and exhibited their characteristic surface plasmonic resonance peak at 576 nm.

In this regard, copper nanoparticles usually exhibit a characteristic surface plasmonic resonance (SPR) peak in the range 560–570 nm; larger particles may cause this resonance peak to be shifted toward longer wavelengths [32]. The exact position of SRP peak may be shifted based on the individual particles properties including the shape, size, capping agent, and the exact chemical composition [33]. Furthermore, the characteristic SPR peak of copper appears also in the presence of small portions of copper oxide at 580 nm [34]. This interprets to a great extent the emergence of the characteristic surface plasmonic resonance peak of the synthesized Cu/Cu_2_O NPs at 576 nm.

### 3.4 Transmission electron microscopy (TEM)

**Figure 4:**
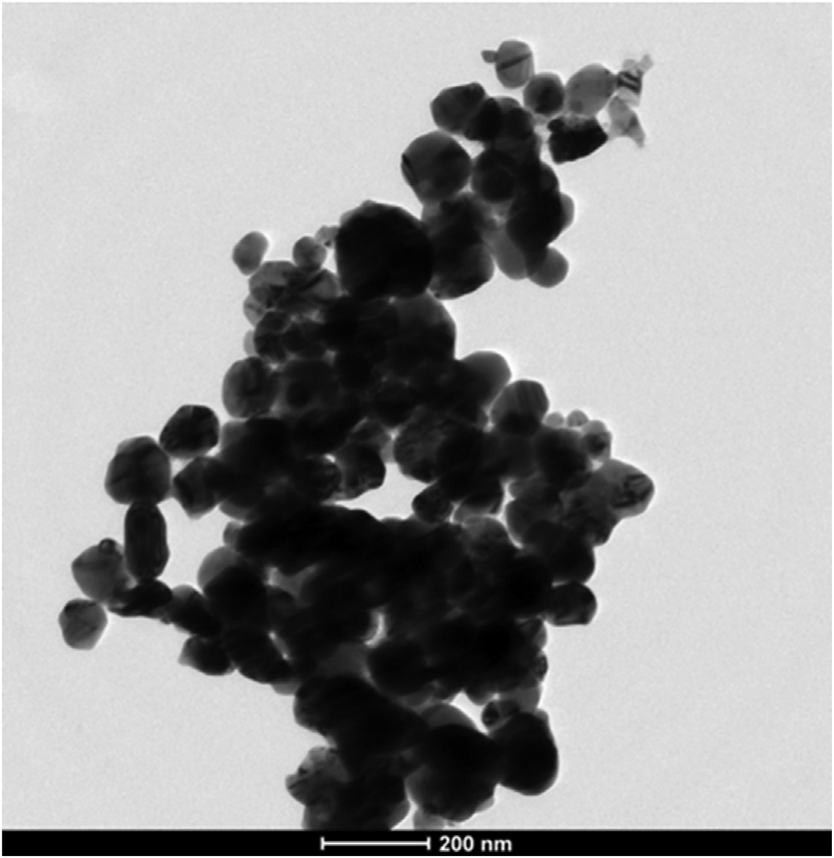
Transmission electron micrograph showing the spherical shape of the synthesized Cu/Cu_2_O NPs.

From transmission electron microscopy, it is clear that the synthesized Cu/Cu_2_O NPs were largely uniform and have a spherical shape, as shown in Figure 4; this result is consistent with the shape and uniformity of Copper/copper oxide nanoparticles [35] and copper nanoparticles [36] synthesized through the green approaches using other plant extracts.

### 3.5 Determining particle size distribution and zeta potential of Cu/Cu_2_O NPs

**Figure 5:**
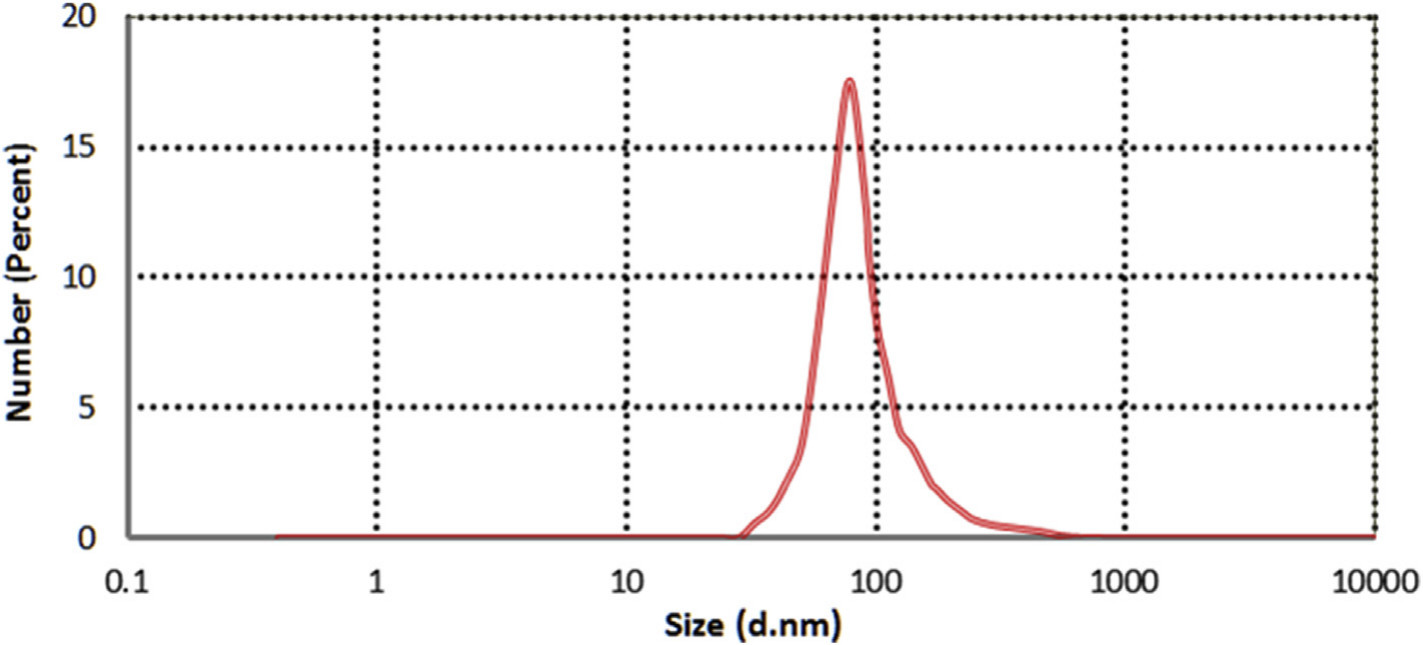
Particle Size Distribution (PSD) of the synthesized Cu/Cu_2_O NPs, showing the mean diameter at 78 nm.

**Figure 6:**
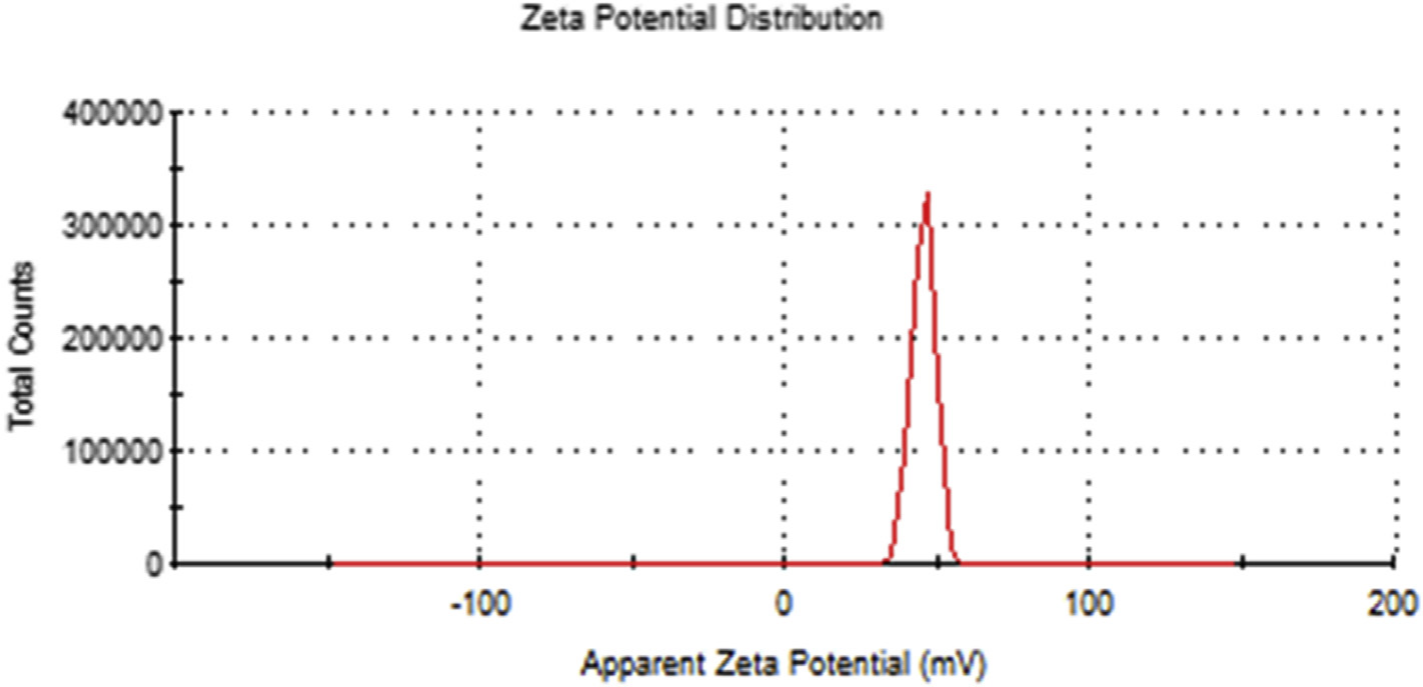
Zeta potential of the synthesized Cu/Cu_2_O NPs at +41 mV.

Dynamic light scattering (DLS) was used to determine both the Particle Size Distribution (PSD) and Zeta Potential (ZP) of the synthesized Cu/Cu_2_O NPs. The results were displayed as an intensity-based Particle Size Distribution and Zeta Potential distribution, as shown in Figures 5 and 6, respectively.

As shown in Figure 5, the average particle size was about 78 nm. This size falls within the specified range for identifying a nanomaterial according to the ***American Society for Testing and Materials* (ASTM)** and the ***International Organization for Standardization* (ISO)**, which is from 1 nm to 100 nm.

As shown in Figure 6, zeta potential of the synthesized Cu/Cu_2_O NPs was +41 mV. In this regard, zeta potential is considered an important indicator for the stability of a colloid, since the magnitude of the zeta potential expresses the extent of the electrostatic repulsion between the same species constituting that colloid. The value of +41 mV indicates that the prepared nanoparticles exhibit a good stability [37]. This good stability of the nanoparticles reported here may be attributed to the collaborative capping effect of both CTAB and the phenolic compounds adsorbed on the surface of the synthesized nanoparticles.

## 4 Conclusion

The increased applicability of copper and copper oxide nanoparticles among different fields, including but not limited to medical, industrial, biological and electronic ones, has encouraged the research for alternative preparation methods, which offer more facile preparation and being more cost-effective. On the other hand, green methods are still attracting more attention and interest due to its simplicity, cost efficiency, nontoxicity and being environment-friendly. In this context, the present paper provides, for the first time, a green chemical reduction method for synthesizing Cu/Cu_2_O NPs using an abundant useless material, the seedless dates, thereby significantly lower the cost of preparation and increase the economic value of the seedless dates through an ecofriendly method. In addition, the current method includes moderate reaction conditions and requires no complex setups, which makes it more cost-effective and increases its feasibility to be applied in a larger scale. Further research is encouraged to assess the efficiency of the proposed method for preparing other metallic nanoparticles.

## Declarations

### Author contribution statement

Elwy A. Mohamed: Conceived and designed the experiments; Performed the experiments; Analyzed and interpreted the data; Contributed reagents, materials, analysis tools or data; Wrote the paper.

### Funding statement

This research did not receive any specific grant from funding agencies in the public, commercial, or not-for-profit sectors.

### Competing interest statement

The author declare no conflict of interest.

### Additional information

No additional information is available for this paper.

## References

[1] Feynman R.P. (1960). There's plenty of room at the bottom.

[2] Lee B., Kim Y., Yang S., Jeong I., Moon J. (2009). A low-cure-temperature copper nano ink for highly conductive printed electrodes. Curr. Appl. Phys..

[3] Ponce A.A., Klabunde K.J. (2005). Chemical and catalytic activity of copper nanoparticles prepared via metal vapor synthesis. J. Mol. Catal. A Chem..

[4] Xu Q., Zhao Y., Xu J.Z., Zhu J.J. (2006). Preparation of functionalized copper nanoparticles and fabrication of a glucose sensor. Sens. Actuators B Chem..

[5] Lin S.C., Al-Kayiem H.H. (2016). Evaluation of copper nanoparticles–Paraffin wax compositions for solar thermal energy storage. Sol. Energy.

[6] Mohamed Elwy A., Gaber Mohamed H., Elsharabasy Sherif F. (2018). Evaluating the in vivo efficacy of copper-chitosan nanocomposition for treating vascular wilt disease in date palm. Int. J. Environ. Agric. Biotechnol..

[7] Mohamed E.A. (2018). Non-Dependency of In Vitro Fungicidal Efficiency of Copper Nanoparticles against Fusarium oxysporum upon Particle Size. J. Plant Pathol. Microbiol..

[8] Mohamed Elwy A., Elsharabasy Sherif F., Abdulsamad Doaa (2019). Evaluation of In vitro Nematicidal Efficiency of Copper Nanoparticles against Root-knot Nematode Meloidogyne Incognita. South Asian J. Parasitol..

[9] Shende S., Ingle A.P., Gade A., Rai M. (2015). Green synthesis of copper nanoparticles by Citrus medica Linn. (Idilimbu) juice and its antimicrobial activity. World J. Microbiol. Biotechnol..

[10] Liu Q.M., Zhou D.B., Yamamoto Y., Ichino R., Okido M. (2012). Preparation of Cu nanoparticles with NaBH4 by aqueous reduction method. Trans. Nonferrous Metals Soc. China.

[11] Su X., Zhao J., Bala H., Zhu Y., Gao Y., Ma S., Wang Z. (2007). Fast synthesis of stable cubic copper nanocages in the aqueous phase. J. Phys. Chem. C.

[12] Zhou R., Wu X., Hao X., Zhou F., Li H., Rao W. (2008). Influences of surfactants on the preparation of copper nanoparticles by electron beam irradiation. Nucl. Instrum. Methods Phys. Res. Sect. B Beam Interact. Mater. Atoms.

[13] Cuevas R., Durán N., Diez M.C., Tortella G.R., Rubilar O. (2015). Extracellular biosynthesis of copper and copper oxide nanoparticles by Stereum hirsutum, a native white-rot fungus from chilean forests. J. Nanomat..

[14] Zhu H.T., Lin Y.S., Yin Y.S. (2004). A novel one-step chemical method for preparation of copper nanofluids. J. Colloid Interface Sci..

[15] Venkatesha N.J., Ramesh S. (2018). Citric acid-assisted synthesis of nanoparticle copper catalyst supported on an oxide system for the reduction of furfural to furfuryl alcohol in the vapor phase. Ind. Eng. Chem. Res..

[16] Biçer M., Şişman İ. (2010). Controlled synthesis of copper nano/microstructures using ascorbic acid in aqueous CTAB solution. Powder Technol..

[17] Sheibani S., Ataie A., Heshmati-Manesh S., Khayati G.R. (2007). Structural evolution in nano-crystalline Cu synthesized by high energy ball milling. Mat. Lett..

[18] Lee H.J., Song J.Y., Kim B.S. (2013). Biological synthesis of copper nanoparticles using Magnolia kobus leaf extract and their antibacterial activity. J. Chem. Technol. Biotechnol..

[19] Gopinath M., Subbaiya R., Selvam M.M., Suresh D. (2014). Synthesis of copper nanoparticles from Nerium oleander leaf aqueous extract and its antibacterial activity. Int. J. Curr. Microbiol. App. Sci..

[20] Kaur P., Thakur R., Chaudhury A. (2016). Biogenesis of copper nanoparticles using peel extract of Punica granatum and their antimicrobial activity against opportunistic pathogens. Green Chem. Lett. Rev..

[21] Khani R., Roostaei B., Bagherzade G., Moudi M. (2018). Green synthesis of copper nanoparticles by fruit extract of Ziziphus spina-christi (L.) Willd.: Application for adsorption of triphenylmethane dye and antibacterial assay. J. Mol. Liq..

[22] Hemmati S., Mehrazin L., Hekmati M., Izadi M., Veisi H. (2018). Biosynthesis of CuO nanoparticles using Rosa canina fruit extract as a recyclable and heterogeneous nanocatalyst for CN Ullmann coupling reactions. Mat. Chem. Phys..

[23] Yugandhar P., Vasavi T., Rao Y.J., Devi P.U.M., Narasimha G., Savithramma N. (2018). Cost effective, green synthesis of copper oxide nanoparticles using fruit extract of Syzygium alternifolium (Wt.) Walp., characterization and evaluation of antiviral activity. J. Clust. Sci..

[24] Thakur S., Sharma S., Thakur S., Rai R. (2018). Green synthesis of copper nano-particles using Asparagus adscendens roxb. Root and leaf extract and their antimicrobial activities. Int. J. Curr. Microbiol. Appl. Sci..

[25] Ehsan M.F., Barakat M.A., Husein D.Z., Ismail S.M. (2014). Immobilization of Ni and Cd in soil by biochar derived from unfertilized dates. Water, Air, Soil Pollut..

[26] Zhu X., Wang B., Shi F., Nie J. (2012). Direct, rapid, facile photochemical method for preparing copper nanoparticles and copper patterns. Langmuir.

[27] Johan M.R., Suan M.S.M., Hawari N.L., Ching H.A. (2011). Annealing effects on the properties of copper oxide thin films prepared by chemical deposition. Int. J. Electrochem. Sci..

[28] Nasrollahzadeh M., Sajadi S.M., Khalaj M. (2014). Green synthesis of copper nanoparticles using aqueous extract of the leaves of Euphorbia esula L and their catalytic activity for ligand-free Ullmann-coupling reaction and reduction of 4-nitrophenol. RSC Adv..

[29] Hassanien R., Husein D.Z., Al-Hakkani M.F. (2018). Biosynthesis of copper nanoparticles using aqueous Tilia extract: antimicrobial and anticancer activities. Heliyon.

[30] Hassanien R., Al-Said S.A.F., Šiller L., Little R., Wright N.G., Houlton A., Horrocks B.R. (2012). Smooth and conductive DNA-templated Cu_2_O nanowires: growth morphology, spectroscopic and electrical characterization. Nanotechnology.

[31] Shoeib M.A., Abdelsalam O.E., Khafagi M.G., Hammam R.E. (2012). Synthesis of Cu_2_O nanocrystallites and their adsorption and photocatalysis behavior. Adv. Powder Technol..

[32] Kaminskienė Ž., Prosyčevas I., Stonkutė J., Guobienė A. (2013). Evaluation of optical properties of Ag, Cu, and Co nanoparticles synthesized in organic medium. Acta Phys. Pol..

[33] Chattopadhyay D.P., Patel B.H. (2012). Preparation, characterization and stabilization of nanosized copper particles. Int. J. Pure Appl. Sci. Technol..

[34] Joseph A.T., Prakash P., Narvi S.S. (2016). Phytofabrication and Characterization of copper nanoparticles using Allium sativum and its antibacterial activity. Int. J. Sci. Eng. Technol..

[35] Khatami M., Heli H., Jahani P.M., Azizi H., Nobre M.A.L. (2017). Copper/copper oxide nanoparticles synthesis using Stachys lavandulifolia and its antibacterial activity. Iet Nanobiotechnol..

[36] Nasrollahzadeh M., Momeni S.S., Sajadi S.M. (2017). Green synthesis of copper nanoparticles using Plantago asiatica leaf extract and their application for the cyanation of aldehydes using K4Fe (CN) 6. J. Colloid Interface Sci..

[37] ÁO'Brien Richard W. (1990). Electroacoustic studies of moderately concentrated colloidal suspensions. Faraday Discuss. Chem. Soc..

